# The June Meeting of the I. H. A.

**Published:** 1884-05

**Authors:** C. Pearson

**Affiliations:** Washington


### THE JUNE MEETING OF THE I. H. A.

In the March number of the PHYSICIAN appears a notice that the next meeting of the Internationals will be held in Washington. As this may be news to many, it might be well to give a brief statement as to how and why this change was made. At the meeting in 1882, a resolution was adopted constituting the officers of the Association an Executive Committee with power to make arrangements for meetings, and to transact such other business pertaining to the interests of the Association as they might deem best. At the meeting last year it was agreed to hold the coming meeting at Deer Park, commencing three days before that of the Institute. Some time after that I received numerous letters from members stating their intention of visiting this city either on their way going to, or returning from, the Deer Park meeting, and suggesting that the place of meeting be changed. I, as the then chairman of the Executive Committee, did not feel disposed to take the responsibility of making the change without consulting the members in attendance at the last meeting, and who had decided to go to Deer Park.

I, therefore, wrote to all the members who were in attendance at that meeting, and received a response from all, and with only two exceptions all voted to have the meeting held in Washington, commencing three or four days prior to meeting of Institute.

I communicated this result to the President-elect, Dr. Foote, who favored the movement, and he will no doubt give official notice of the time as soon as it is ascertained on what days of June the Institute meets. The President has also appointed me a committee *of one* to make arrangements for place of meeting, hotel accommodations, etc. This will be attended to, and as the hotel fare at the Park is to be $2.50 per day, I am disposed to think it will not exceed that here, but of this I will give further notice in the June number of the Journals.* Our city at that time will be looking its best, and as Congress will then be in session, it is to be hoped that every member who can possibly do so will be in attendance. And not only do we desire members to attend, but those who are not; we are not afraid of being contaminated, since the Institute has failed to do this, while our rigid homœopathic prescribing may do them much good. All are welcome.

C. PEARSON.

---

* The meeting will be held at 611 Twelfth Street, Washington, June 13th, at 10 A. M. For information as to board, etc., communicate with Dr. Pearson.—EDITOR.

